# PI3Kγ Protects from Myocardial Ischemia and Reperfusion Injury through a Kinase-Independent Pathway

**DOI:** 10.1371/journal.pone.0009350

**Published:** 2010-02-22

**Authors:** Bernhard J. Haubner, G. Gregory Neely, Jakob G. J. Voelkl, Federico Damilano, Keiji Kuba, Yumiko Imai, Vukoslav Komnenovic, Agnes Mayr, Otmar Pachinger, Emilio Hirsch, Josef M. Penninger, Bernhard Metzler

**Affiliations:** 1 Department of Internal Medicine III (Cardiology), Innsbruck Medical University, Innsbruck, Austria; 2 Institute of Molecular Biotechnology of the Austrian Academy of Sciences, Vienna, Austria; 3 Department of Genetics, Biology and Biochemistry, Molecular Biotechnology Center, University of Torino, Torino, Italy; Instituto de Química, Universidade de São Paulo, Brazil

## Abstract

**Background:**

PI3Kγ functions in the immune compartment to promote inflammation in response to G-protein-coupled receptor (GPCR) agonists and PI3Kγ also acts within the heart itself both as a negative regulator of cardiac contractility and as a pro-survival factor. Thus, PI3Kγ has the potential to both promote and limit M I/R injury.

**Methodology/Principal Findings:**

Complete PI3Kγ^−/−^ mutant mice, catalytically inactive PI3Kγ^KD/KD^ (KD) knock-in mice, and control wild type (WT) mice were subjected to *in vivo* myocardial ischemia and reperfusion (M I/R) injury. Additionally, bone-marrow chimeric mice were constructed to elucidate the contribution of the inflammatory response to cardiac damage. PI3Kγ^−/−^ mice exhibited a significantly increased infarction size following reperfusion. Mechanistically, PI3Kγ is required for activation of the Reperfusion Injury Salvage Kinase (RISK) pathway (AKT/ERK1/2) and regulates phospholamban phosphorylation in the acute injury response. Using bone marrow chimeras, the cardioprotective role of PI3Kγ was mapped to non-haematopoietic cells. Importantly, this massive increase in M I/R injury in PI3Kγ^−/−^ mice was rescued in PI3Kγ kinase-dead (PI3Kγ^KD/KD^) knock-in mice. However, PI3Kγ^KD/KD^ mice exhibited a cardiac injury similar to wild type animals, suggesting that specific blockade of PI3Kγ catalytic activity has no beneficial effects.

**Conclusions/Significance:**

Our data show that PI3Kγ is cardioprotective during M I/R injury independent of its catalytic kinase activity and that loss of PI3Kγ function in the hematopoietic compartment does not affect disease outcome. Thus, clinical development of specific PI3Kγ blockers should proceed with caution.

## Introduction

Cardiac damage in acute myocardial infarction results from a disruption of blood flow to the heart (ischemia) that may be followed by either spontaneous or therapeutically induced restoration of blood supply (reperfusion). Myocardial ischemia/reperfusion (M I/R) results in infarction due to intrinsic cardiomyocyte death [1], [2] and damage via excessive immune activation [3], [4], [5]. Many inflammatory factors that build up during injury exert their effects through G-protein coupled receptors (GPCRs) [6] suggesting that mediators of GPCRs might be critically involved in the M I/R damage response. One key signaling pathway downstream of GPCRs is mediated via phosphoinositide 3-kinase γ (PI3Kγ).

Phosphatidylinositol-3-OH kinases (PI3K) are lipid kinases that convert phosphatidylinositol 4,5-bisphosphate (PIP2) into phosphatidylinositol (3,4,5)-trisphosphate (PIP3). In mammals, there are four class I PI3K, three of which (α, β, and δ) are primarily activated by tyrosine kinase signaling pathways, whereas PI3Kγ is the principal PI3K that relays signals via GPCRs to downstream pathways such as AKT and ERK/1/2 [7]. PI3Kγ^−/−^ mice (lacking the catalytic subunit p110) are viable and in certain experimental settings exhibit reduced inflammation [8], [9] as the PI3Kγ pathway controls migration of inflammatory cells [10]. In addition, we have previously shown that PI3Kγ acts as an intrinsic negative regulator of cardiac contractility [11], [12]. Given its dual roles in cardiomyocyte function and inflammation, it is not clear if the net effect of PI3Kγ loss is cardio-protective or cardio-destructive during M I/R. For example, we have previously shown that PI3Kγ^−/−^ mice are resistant to the detrimental effects of chronic β-adrenergic receptor signaling [13]. Conversely, PI3Kγ^−/−^ mice showed a worse outcome both in a model of chronic myocardial ischemia without reperfusion [14] and chronic pressure overload [12], while overexpression of catalytically inactive PI3Kγ protected from heart failure in this model [15]. Moreover, it has been reported that a PI3Kγ blocker might protect from M I/R injury due to inhibition of the inflammatory response during reperfusion [16]. However, since such blockers are not specific for PI3Kγ and PI3Kγ inhibitors are currently developed as a novel treatment option for M I/R in humans, it is paramount to genetically define the role of PI3Kγ in cardiac ischemia/reperfusion injury.

We therefore assessed the role of PI3Kγ in M I/R injury using PI3Kγ^−/−^ mice. We report that PI3Kγ mutant mice develop massive early and late stage cardiac M I/R injury. PI3Kγ seems to be the key kinase that activates the Reperfusion Injury Salvage Kinase (RISK) pathway (AKT/ERK1/2) in the acute setting of M I/R. Using bone marrow chimeras these effects were mechanistically mapped to non-haematopoetic cells suggesting that the effects are intrinsic to cardiac tissue. To assess whether these effects are dependent on catalytic activity, we performed M I/R injury in mice with a knock-in mutation in the PI3Kγ kinase domain (PI3Kγ^KD/KD^). PI3Kγ^KD/KD^ mice developed cardiac injury similar to wild type mice. Thus, PI3Kγ protects the heart from M I/R injury through a kinase-independent mechanism *in vivo*.

## Results

### Genetic Inactivation of PI3Kγ Results in Severe Cardiac Damage to M I/R Injury

**Figure 1:**
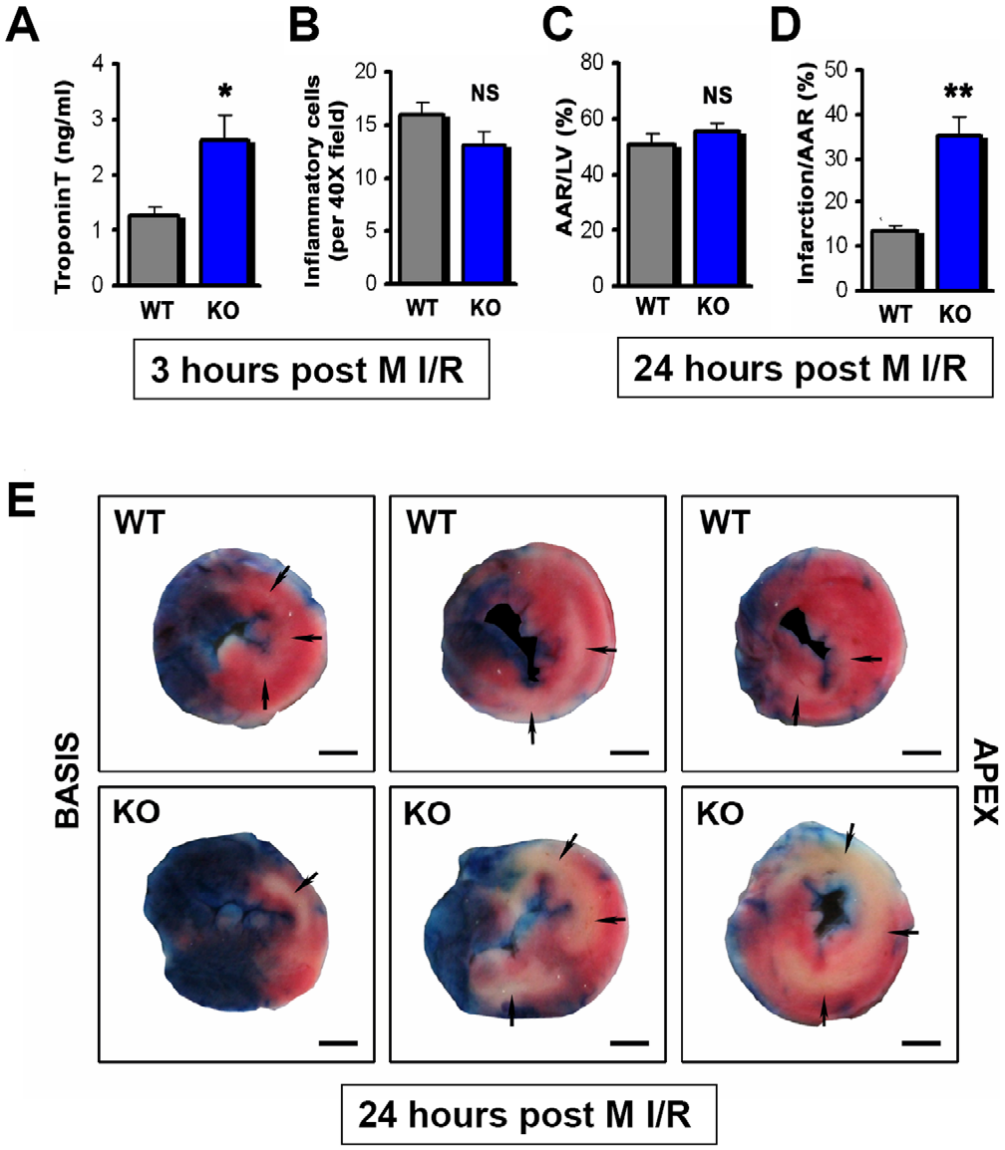
Loss of PI3Kγ results in massive myocardial ischemia/reperfusion induced heart damage. (**A**) Following M I/R injury, serum TroponinT is significantly increased in PI3Kγ^−/−^ (KO) mice compared to wild type (WT) controls. Data are from 3 hours after reperfusion. n = 13 per group. (**B**) No significant difference in the numbers of inflammatory cells in the injured hearts of PI3Kγ^−/−^ and WT mice. Inflammatory cell infiltrates were assessed 3 hours after reperfusion. n = 7 per group. (**C**) No significant difference in the area at risk (AAR)/left ventricle (LV) in PI3Kγ^−/−^ and WT mice. (**D**) PI3Kγ KO mice display a markedly increased area of infarction/area at risk (AAR) 24 hours after reperfusion. n = 6 per group for (**C**) and (**D**). (**E**) Representative left ventricular TTC-stained sections from the basis (left) to the apex (right) of wild type (WT; upper panels) and PI3Kγ^−/−^ (KO; lower panels) hearts after 24 hours of reperfusion. Pale areas represent necrotic regions (arrows). Bars indicate 1 mm. In all bar graphs mean values +/− SEM are shown. *p<0.05. **p<0.01. NS = not significant.

To assess the role of PI3Kγ in M I/R injury, we performed *in vivo* reversible LAD ligation in sex and age matched PI3Kγ^−/−^ (KO) mice and PI3Kγ expressing wild type mice (WT). The extent of injury and heart functions were assayed 3 hours, 24 hours, 1 week, and 3 weeks after initiation of reperfusion. Intriguingly, in PI3Kγ^−/−^ mice serum Troponin T levels, a marker of cardiac damage, were significantly increased after 3 hours of reperfusion (Figure 1A), without a significant change in the number of inflammatory cells infiltrating the damaged cardiac tissue (Figure 1B). In parallel, we observed a massive increase of the infarction size within the area at risk in PI3Kγ^−/−^ hearts at the early stage of M I/R injury (assayed at 24 hours, Figure 1C–E). Thus, immediately after M I/R the loss of PI3Kγ expression results in massive early heart damage.

**Figure 2:**
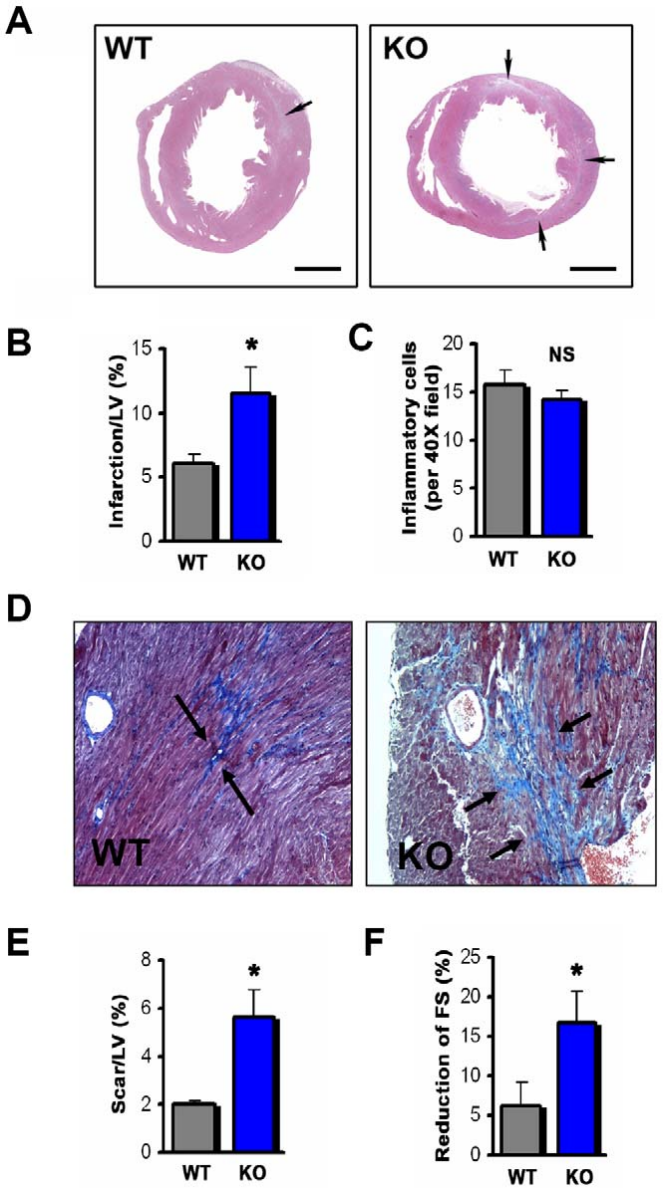
Loss of PI3Kγ results in long term myocardial ischemia/reperfusion induced heart damage. (**A**) Areas of infarction (arrows) in representative left mid-ventricular sections 1 week after M I/R injury. (H&E staining). Bars indicate 1 mm. (**B**) PI3Kγ^−/−^ (KO) hearts show a markedly greater infarction size in the left ventricle (LV) at 1 week after M I/R. n = 7 per group. (**C**) No significant difference between PI3Kγ^−/−^ and WT mice in the numbers of infiltrating inflammatory cells 1 week after M I/R. n = 7 per group. (**D**) Representative left mid-ventricular sections stained with Trichrome and Aniline blue to visualize collagen deposits (arrows) in hearts of PI3Kγ^−/−^ (KO) and wild type (WT) mice 3 weeks after M I/R. Magnifications ×20. (**E**) Markedly increased scar tissue in PI3Kγ^−/−^ 3 weeks after M I/R injury. n = 7 per group. (**F**) PI3Kγ^−/−^ mice exhibit a significant greater loss in percentage fractional shortening (FS) relative to their respective baseline FS after 3 weeks of M I/R compared to WT controls. n = 9 per group. In all bar graphs mean values +/− SEM are shown. *p<0.05. NS = not significant.

It was possible that in the long term the early cardioprotective effects of PI3Kγ would not outweigh secondary pro-inflammatory effects due to impaired migration of inflammatory cells into the damaged heart; therefore, the net result of disrupting PI3Kγ expression could be cardioprotective. To address this possibility we performed M I/R injury on wild type (WT) and PI3Kγ^−/−^ (KO) mice and followed these mice for up to three weeks. One week after M I/R injury, PI3Kγ^−/−^ mice still exhibited a massive increase in infarction size as compared to the control cohort (Figure 2A,B). Again we observed no significant differences in the numbers of inflammatory cells infiltrating the cardiac tissue between control and PI3Kγ^−/−^ mice (Figure 2C), suggesting that PI3Kγ is not required for recruitment of inflammatory cells to the infarction site during the early (3 hours) as well as later stages (1 week) stages of M I/R injury.

Following the inflammatory phase of M I/R injury, scar formation occurs at the infarction site. In both wild type and PI3Kγ^−/−^ mice the primary damage site was replaced by scar tissue 3 weeks after the initial M I/R injury (Figure 2D). In line with the increased injury, collagen deposition and scar formation was markedly increased in PI3Kγ^−/−^ mice (Figure 2D, E). We next assessed cardiac function 3 weeks after M I/R injury. Functionally, the excessive early damage and increased scar formation in M I/R injured PI3Kγ^−/−^ mice culminated in a severe reduction in cardiac contractility as defined by changed fractional shortening from the baseline (Figure 2F). Thus, PI3Kγ is cardio-protective during M I/R injury and genetic disruption of PI3Kγ results in severe cardiac damage that is sustained over time and severely compromises cardiac function.

### PI3Kγ Affects Cardiac M I/R Injury Independent of the Haematopoietic System

**Figure 3:**
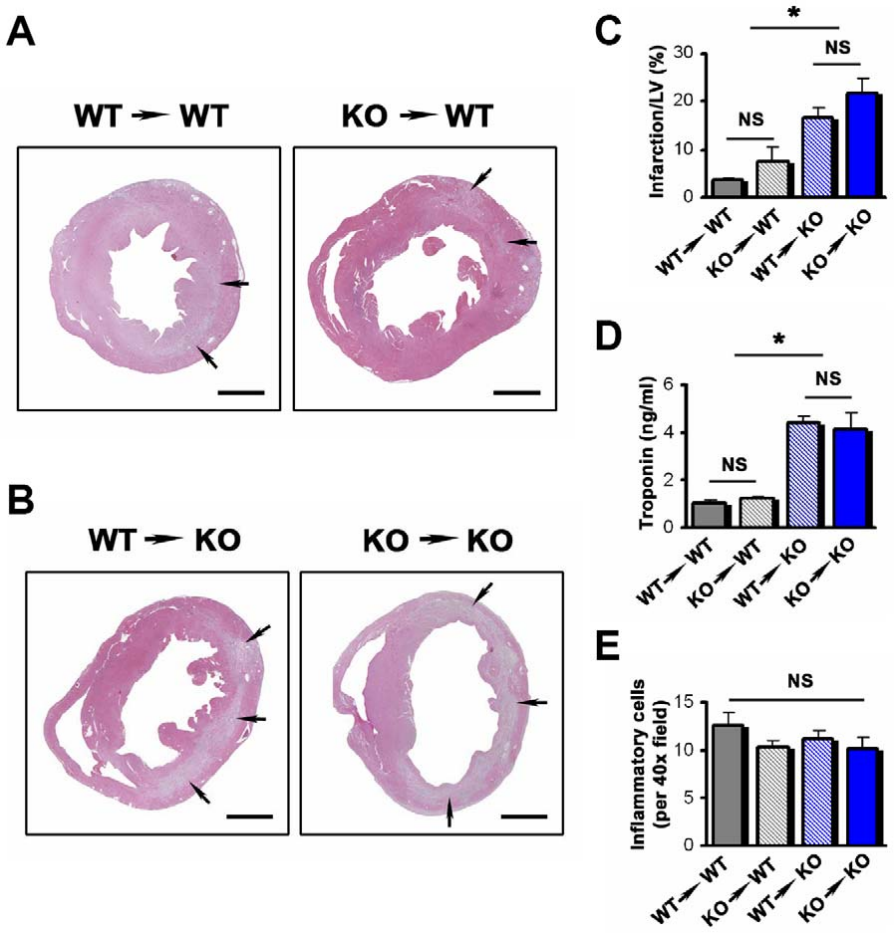
PI3Kγ functions in non-haematopoietic cells in myocardial ischemia/reperfusion injury. (**A**) Representative left mid-ventricular histological sections (H&E staining) from WT mice receiving WT bone marrow (WT→WT) and WT mice receiving PI3Kγ^−/−^ bone marrow (KO→WT). Hearts were isolated and analysed from mice 1 week after M I/R injury. Arrows indicate areas of infarction. (**B**) Representative left mid-ventricular histological sections (H&E staining) from PI3Kγ^−/−^ mice receiving WT bone marrow (WT→KO) and PI3Kγ^−/−^ mice receiving PI3Kγ^−/−^ bone marrow (KO→KO) after 1 week of M I/R injury. Arrows point at areas of infarction. (**C**) No significant difference between WT→WT and KO→WT chimeras and no difference between WT→KO and KO→KO chimeras in the size of infarction determined 1 week after M I/R injury. n = 5 per group. (**D**) No significant difference between WT→WT and KO→WT chimeras and no difference between WT→KO and KO→KO chimeras in TroponinT levels assayed 3 hours after reperfusion. n = 7 per group. (**E**) No significant difference between all 4 groups of chimeric mice in the numbers of infiltrating inflammatory cells 1 week after M I/R. n = 5 per group. In all histological pictures the bar indicates 1 mm. All bar graphs show mean values +/− SEM. *p<0.05. NS = no significant difference.

PI3Kγ controls multiple activation pathways in haematopoetic cells including cell migration [7] and it has been proposed that PI3Kγ might be a key drug target to block inflammation in various pathologic conditions including M I/R injury in heart attacks [17]. To directly test whether the protective effects of PI3Kγ are due to its function in the immune system, we generated bone marrow chimeras. Interestingly, although PI3Kγ has clearly been implicated in inflammation in many other systems [7], wild type mice given either wild type or PI3Kγ^−/−^ bone marrow developed comparable M I/R injuries (Figure 3A, C) and showed equal Troponin T release (Figure 3D). Moreover, PI3Kγ^−/−^ and wild type immune cells accumulated to a similar extent at the site of M I/R injury (Figure 3E).

In the reciprocal experiment, wild type or PI3Kγ^−/−^ bone marrow was transferred into PI3Kγ^−/−^ recipients; in these bone marrow chimeras we again observed comparable infarction sizes (Figure 3B, C) and Troponin T release (Figure 3D). Irrespective of the genotype of the transferred bone marrow cells, PI3Kγ^−/−^ recipient mice exhibited similar inflammation at the site of infarction (Figure 3E). Of note, in all bone marrow transplantation experiments, PI3Kγ^−/−^ recipient mice developed markedly more severe cardiac damage following M I/R injury as compared to wild type host mice (Figure 3B–D). These data indicate that the excessive damage seen in PI3Kγ^−/−^ mice following M I/R injury occurs independent of its role in haematopoietic cells.

### Loss of PI3Kγ Results in Impaired RISK Signaling

**Figure 4:**
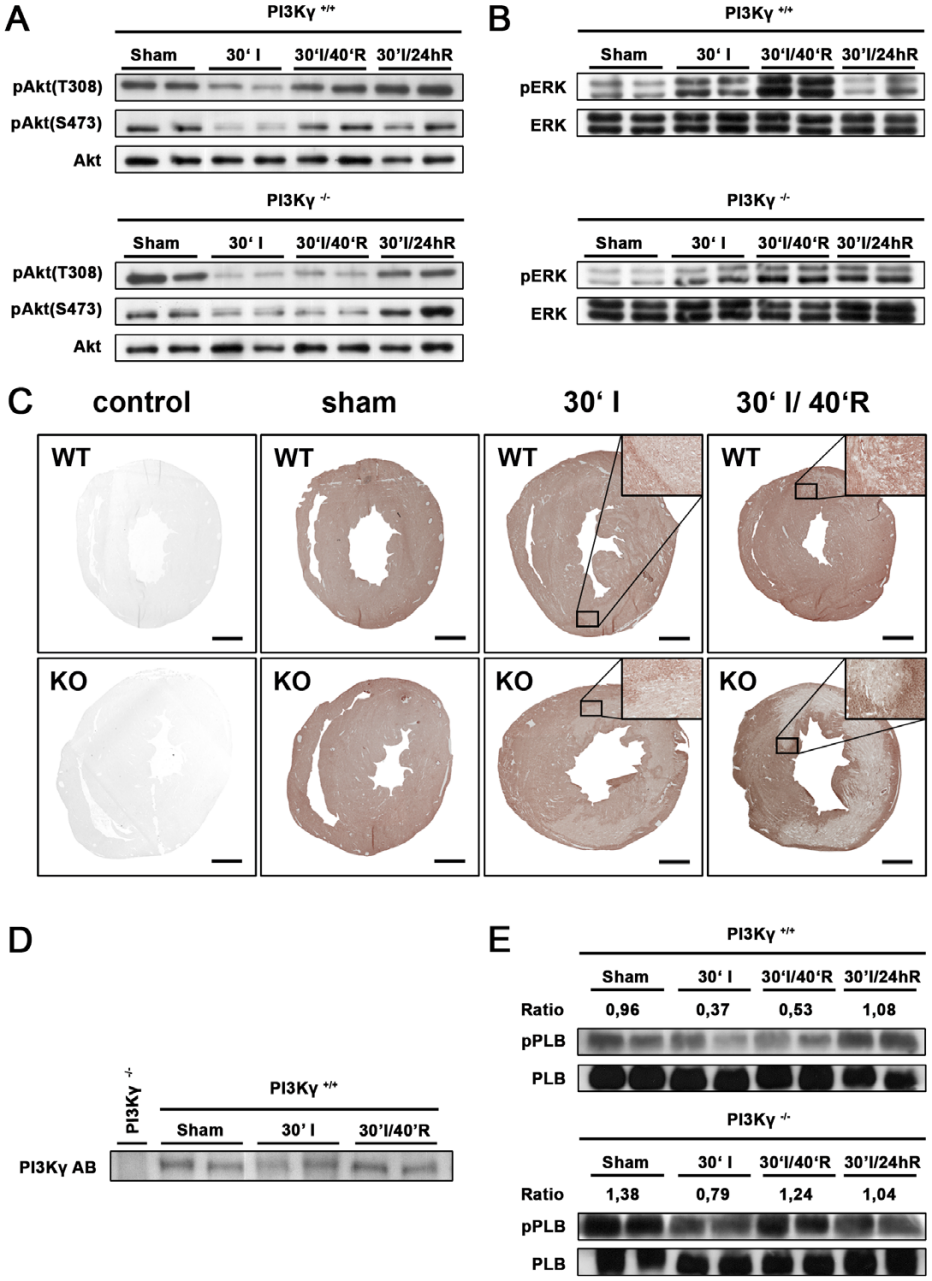
PI3Kγ is the critical mediatior of AKT and ERK1/2 activation in the acute phase of ischemia/reperfusion injury. (**A**) Western blot analysis of phosphorylated AKT (T308 and S473) and (**B**) phosphorylated ERK1/2 (T202/Y204) in PI3Kγ^−/−^ and wild type PI3Kγ^+/+^ hearts after sham surgery, 30 minutes of ischemia (30'I), 30 minutes of ischemia plus 40 minutes of reperfusion (30'I/40'R), and 30 minutes of ischemia plus 24 hrs of reperfusion (30'I/24hR). Total AKT and ERK1/2 levels are shown as controls. Representative data from 2 mice for each genotype and treatment protocol are shown. (**C**) Immunohistochemical staining for phosphorylated AKT (S473) in PI3Kγ^−/−^ (KO) and wild type (WT) hearts after sham surgery (left panels), 30 minutes of ischemia (30'I), and 30 minutes of ischemia plus 40 minutes of reperfusion (30'I/40'R). A secondary antibody staining control is shown (control). Bar indicates 1 mm. Inserts are 20× magnifications. (**D**) Western blot analysis of wild type hearts to determine PI3Kγ expression levels in sham-treated mice, 30 minutes of ischemia (30'I), and 30 minutes of ischemia plus 40 minutes of reperfusion (30'I/40'R). Representative data from 2 mice for each genotype and treatment protocol are shown. Heart lysates from PI3Kγ^−/−^ mice are shown as a specificity control. (**E**) Increased phosphorylated phospholamban B (pPLB)/phospholamban B (PLB) ratios in hearts from PI3Kγ^−/−^ mice compared to wild type mice after sham surgery, 30 minutes of ischemia (30'I), 30 minutes of ischemia plus 40 minutes of reperfusion (30'I/40'R), and 30 minutes of ischemia plus 24 hrs of reperfusion (30'I/24hR). Western blot results are shown as duplicates. For all Western blots, the area at risk was dissected from the left ventricle.

Activation of the PI3K pathway leads to cell survival through activation of the Reperfusion Injury Salvage Kinase (RISK) pathway (AKT/ERK1/2) [2], [10]. Similar to the growth factor coupled class IA PI3Ks α,β, and δ, PI3Kγ can relay GPCR signals to activate AKT and ERK1/2 in cardiomyocytes [11], [12], [13]. To assess AKT and ERK activation in M I/R injury, we isolated hearts from wild type and PI3Kγ^−/−^ mice prior to ischemia, after 30 minutes of ischemia, and 40 minutes as well as 24 hours after initiation of reperfusion. Protein extracts were isolated from the area at risk and immunoblotted for phosphorylated AKT (T308 and S473, Figure 4A) and phosphorylated ERK1/2 (T202/Y204, Figure 4B), both indicative of activate AKT and ERK1/2.

As previously reported [11], [13], wild type and PI3Kγ^−/−^ mice exhibit comparable baseline phosphorylation of both AKT and ERK1/2. Following 30 minutes of ischemia due to *in vivo* LAD ligation, AKT and ERK1/2 activation were comparably reduced in both genotypes. Ischemia-induced decrease in AKT and ERK1/2 phosphorylation was rapidly restored in wild type animals within 40 minutes of reperfusion. By contrast, in PI3Kγ^−/−^ mice AKT and ERK1/2 phosphorylation were still markedly reduced 40 minutes after reperfusion (Figure 4A, B). These marked differences in AKT and ERK1/2 activation were not apparent after 24 hours of reperfusion, indicating a role of PI3Kγ in the early initiation of pro-survival signaling through AKT and ERK1/2. Immunohistochemical staining to detect phosphorylated AKT (S473) showed that active AKT is localized to the area at risk of wild type hearts (Figure 4C). Of note, *in vivo* M I/R did not alter the PI3Kγ protein content in the early phase of reperfusion (Figure 4D). Thus, PI3Kγ^−/−^ mice fail to activate AKT and ERK1/2 immediately following M I/R injury, and this lack of activation can be localized to the area at risk.

In addition to AKT and ERK1/2 activation, PI3Kγ is a regulator of myocardial contractility [11] via regulation of phosphodiesterases and subsequent phospholamban (PLB) phosphorylation in the sarcoplasmatic reticulum [12], [18]. Phosphorylation of PLB results in increased contractility [11]. Confirming previous results, PI3Kγ^−/−^ hearts exhibit a much higher basal level of phosphorylated PLB (Figure 4E). Whereas in wild type hearts phosphorylated PLB was decreased following ischemia and 40 minutes of reperfusion, such decrease in phosphorylated PLB was less pronounced in ischemic PI3Kγ^−/−^ hearts. Moreover, as compared to wild type control mice, in PI3Kγ^−/−^ mice phosphorylation of PLB was restored to basal levels during the vulnerable early stage (40 minutes) of reperfusion. Thus, loss of PI3Kγ results in marked dysregulation of the RISK pathway and phospholamban.

### Kinase Dependency of PI3Kγ during M I/R

**Figure 5:**
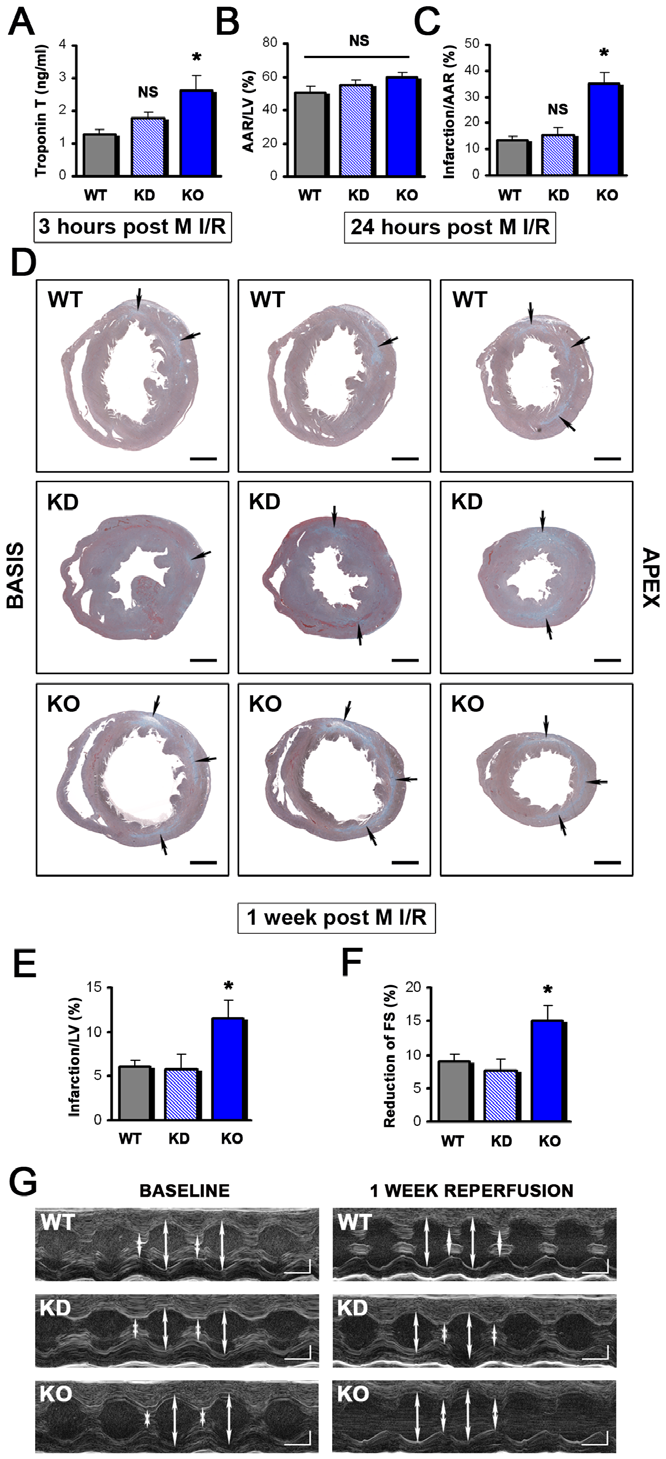
PI3Kγ^−/−^ lipid kinase activity is not required for cardioprotection. (**A**) TroponinT levels measured after 30 minutes of ischemia and 3 hours of reperfusion are comparable between PI3Kγ^KD/KD^ (KD) and control wild type (WT) mice. In total PI3Kγ knock-out (KO) mice TroponinT levels are significantly increased (*p<0.05). n = 13 for WT and KO, n = 11 for KD mice. (**B**) No significant difference in the area at risk (AAR)/left ventricle (LV) in PI3Kγ^−/−^ (KO), PI3Kγ^KD/KD^ (KD), and control WT mice. Data are at 24 hours after M/I injury. (**C**) PI3Kγ KO, but not PI3Kγ^KD/KD^ (KD) mice display a markedly increased area of infarction/area at risk (AAR) 24 hours after reperfusion evaluated by TTCstaining. n = 6 per group. *p<0.05. NS = not significant. (**D**) Representative left ventricular Trichrome-stained sections from the basis (left) to the apex (right) of hearts from wild type (WT), PI3Kγ^KD/KD^ knock-in (KD), and PI3Kγ^−/−^ (KO) mice challenged with 30 minutes of ischemia plus 1 week of reperfusion. Arrows represent infarcted regions. Bars indicate 1 mm. (**E**) PI3Kγ^KD/KD^ (KD) mice display similar infarctions/left ventricle (LV) compared to wild type controls after 30 minutes of ischemia and 1 week of reperfusion. Data from PI3Kγ^−/−^ (KO) are also shown. n = 6 per group. (**F**) PI3Kγ^−/−^ (KO), but not PI3Kγ^KD/KD^ knock-in (KD) mice exhibit a significant greater impairment in percentage fractional shortening (FS) relative to WT controls after 30 minutes of ischemia and 1 week of reperfusion. n = 6 per group. Note that the data are calculated to baseline FS of the respective genotype. All bar graphs show mean values +/− SEM. *p<0.05. (**G**) Representative M-mode echocardiography of wild type (WT), PI3Kγ^KD/KD^ knock-in (KD), and PI3Kγ^−/−^ (KO) mice one week after M I/R injury. Note, that baseline contractility is enhanced in PI3Kγ^−/−^ (KO), but not PI3Kγ^KD/KD^ knock-in (KD) mice as reported previously [11], [12]. Bars indicate 50 ms (horizontal) and 1 mm (vertical).

PI3Kγ exerts kinase-dependent and kinase-independent functions [12]. To address the issue of kinase dependency we subjected wild type, PI3Kγ^−/−^ mice, and PI3Kγ kinase-dead knock-in mice (PI3Kγ^KD/KD^) to M I/R injury. Whereas PI3Kγ^−/−^ mice again exhibited severe infarction following M I/R injury, PI3Kγ^KD/KD^ mice displayed cardiac damage that was comparable to wild type control mice (Figure 5A–E). These differences between PI3Kγ^−/−^ mice and PI3Kγ^KD/KD^ as well as control mice were already evident at 3 and 24 hours of reperfusion (Fig. 5A–C). PI3Kγ^−/−^ mice again showed severely compromised cardiac contractility and fractional shorting following M I/R injury. By contrast, the observed reduction in M I/R-induced infarction size in PI3Kγ^KD/KD^ mice translated into impaired cardiac contractility that was again comparable to that of wild type mice (Figure 5F, G). Thus, the cardioprotective function of PI3Kγ during M I/R injury appears to be independent of its kinase activity. However, PI3Kγ^KD/KD^ mice exhibited a cardiac injury similar to wild type animals.

## Discussion

In the current study we surprisingly observed little requirement for PI3Kγ during inflammation associated with M I/R injury. Genetic evidence has implicated PI3Kγ as a pro-inflammatory intracellular signaling component important for neutrophil chemotaxis [8], [9]. *In vivo*, PI3Kγ^−/−^ mice have been shown defective in diverse models of inflammation, including acute peritonitis [8], [9] and delayed type hypersensitivity [19]. In addition, PI3Kγ^−/−^ mice develop attenuated atherosclerosis [20]. While loss of PI3Kγ alone may slow neutrophil migration [21], this loss does not appear to affect inflammation even in the early stages of our M I/R injury model. It has been shown that *in vivo* PI3Kγ cooperates with PI3Kδ to promote efficient neutrophil migration in response to chemokines and pro-inflammatory cytokines such as TNF-α [22]. Most importantly, our bone marrow transplantation experiments exclude a requirement for PI3Kγ in cardiac inflammation following M I/R injury.

Enhanced contractility by increased PLB phosphorylation [23], [24] and/or reduced RISK signaling via AKT and ERK1/2 [2] could contribute to the severe injury phenotype we observed in PI3Kγ^−/−^ mice. We found that knock-in mice lacking the PI3Kγ kinase activity (PI3Kγ^KD/KD^) display an M I/R injury phenotype that is similar to wild type animals. Therefore, the detrimental outcome observed in PI3Kγ^−/−^ animals appears to be due to kinase-independent events, possibly via phospholamban hyperphospholryation and hypercontractility. PI3Kγ blockers have entered clinical development for chronic inflammation and heart attacks in humans [16], [25]. Our data on PI3Kγ in M I/R injury suggest that specific inhibition of PI3Kγ will not result in any overall improvement concerning cardiac function and the extent of injury. Moreover, we observed a loss of RISK activation in PI3Kγ^−/−^ mice, suggesting that treatment with PI3Kγ blockers may interfere with these early pro-survival signals in some contexts. Of note, the observed loss of immediate RISK activation itself does not appear to compromise M I/R outcome in our model. These data are consistent with other recent studies questioning the cardioprotective role of RISK [26], [27]. Similar to our first in vivo evidence on M I/R injury using mutant mice and kinase-dead knock-in mice, previous work has shown that PI3Kγ is an important signaling pathway that controls ischemic preconditioning *in vitro* [28], arguing that if anything, strategies to activate, not inhibit, PI3Kγ may improve outcome for patients with ischemic heart disease.

The present study provides evidence that PI3Kγ is cardioprotective *in vivo* in a mouse model of myocardial ischemia/reperfusion. Surprisingly, the severe cardiac damage observed in PI3Kγ^−/−^ mice occurs very rapidly and is not influenced by PI3Kγ expression within the hematopoietic compartment, suggesting that PI3Kγ exerts a cell autonomous protective function in cardiomyocytes. Using kinase dead knock-in mice, these detrimental effects were mapped to be independent of PI3Kγ lipid kinase activity. However, loss of PI3Kγ catalytic activity does not translate into a beneficial outcome but could negatively influence RISK signaling during the acute phase of reperfusion. In conclusion, our genetic data indicate that specific inhibition of PI3Kγ is not cardioprotective. Therefore, treatment of acute infarctions with PI3Kγ inhibitors should proceed with caution.

## Methods

### Mice/Ethics Statement

PI3Kγ^−/−^ (p110γ^−/−^) and kinase-dead knock-in PI3Kγ^KD/KD^ mice have been previously described [9], [12] and were backcrossed onto a C57BL/6 background for at least 8 generations. Only male animals were used for the experiments. Animals were handled in accordance with institutional guidelines all experiments were approved by the Austrian Federal Ministry of Science and Research (GZ: BMBWK 66.011/0061BrGT/2006), and in accordance with the Guide for the Care and Use of Laboratory Animals published by the US National Institutes of Health (NIH Publication No. 85–23, revised 1996).

### Surgical Procedure and Experimental Protocol

PI3Kγ^−/−^, PI3Kγ^KD/KD^, and control mice were randomised into a three-hour, 24-hour, one-week, and three-week reperfusion groups and underwent *in vivo* reversible LAD ligation as described [29], [30]. Briefly, following sedation, tracheotomy, ventilation, and thoracotomy, the LAD was reversibly ligated aided by a PE tube. After 30 minutes of ischemia, the PE tube was removed and reperfusion was visually confirmed. The overall peri- and post-procedural mortality was below 10% (control mice: 5,7%; PI3Kγ^−/−^: 6,1%; PI3Kγ^KD/KD^: 9,7%).

### Infarct Evaluation

Heparinized blood was collected from the retrobulbar vein plexus. Plasma levels of troponinT were evaluated as a biomarker for cardiac damage using a quantitative assay (Roche Diagnostics, Austria). Moreover, hearts were fixed in 4% formaldehyde overnight and embedded in paraffin. Three 5-µm sections were cut from defined levels of the left ventricle, stained with hematoxylin and eosin (H&E). Infarct sizes were analyzed using Axio Imager M1 (Zeiss, Axiovert, Jena, Germany) and TissueFAXS software package (TissueGnostics, Vienna, Austria). In addition, formalin-fixed heart sections were stained with Trichrome and Aniline Blue to detect differences in collagen deposition and scar size. Using a light microscope (Axioskop2 equipped with AxioCam MRc5, Zeiss), Axio Vision 4.0 (Zeiss), and Adobe Photoshop CS3, the mean values of inflammatory infiltrate were determined within 3×40 visual fields of each area of infarction. To evaluate the size of the infarction in relation to the area at risk (AAR) and left ventricle size, we performed 2,3,5-triphenyltetrazolium chloride (TTC) staining: The LAD was firmly re-occluded after 3 hours of reperfusion and 0.5 ml of Evan's blue was injected into the left ventricle. The presence of Evan's blue indicates perfusion, whereas its absence indicates lack of perfusion [31]. Sections from the left ventricles were put into a 2% TTC solution (Sigma) and stained for 15 minutes under constant shaking. Whereas brick red areas indicate viable myocardium, white regions demarcate tissue injury. Left ventricle size (LV), area at risk (AAR) and infarct area (Inf) were assessed by a blinded investigator. Measurements from multiple heart slices were averaged and Inf/AAR and AAR/LV calculated.

### Echocardiography

Myocardial contractility was determined by transthoracic echocardiography using a sector scanner (AcuNav 10MHz, Acuson Sequoia C256, Acuson Corporation Mountain View, CA) and Visualsonics Vevo 770. Short-axis 2D-targeted M-mode images of the left ventricle were obtained. Fractional shortening (FS) was calculated from digital images by using a standard formula.

### Bone Marrow Transplantation

Six to eight week-old recipient mice underwent a lethal total-body irradiation (1000 Rad). Freshly isolated total host bone marrow cells were then injected into the lateral tail vein of syngenic recipient mice (5×10^6^ cells per mouse) 24 hours after irradiation. PCR analyses of blood cells and tail DNA indicated that ∼95% of circulating leukocytes were of donor origin.

### Preparation of Cardiac Samples and Immunoblot Analysis

Hearts were quickly removed and the area at risk from the left ventricle was immediately snap-frozen in liquid nitrogen. Proteins were extracted using T-PER Tissue Protein Extraction Reagent containing a Halt Protease and Phosphatase Inhibitor Cocktail (PIERCE, Rockford, USA). Protein extracts (20µg) were subjected to Western blot analysis with antibodies against phospho-ERK (T202/Y204, Cell Signaling), ERK (Cell Signaling), phospho-PLB (S16, Upstate), PLB (clone A1; Upstate), phospho-AKT (S473, R&D Systems), phospho-AKT (T308, Cell Signaling), and AKT (Cell Signaling). For Western blot analysis of PI3Kγ, 40µg of protein was used.

### Immunohistochemistry

Serial 5µm sections were cut from cryo-preserved tissue blocks, fixed in ice-cold acetone for 10 minutes and washed with TRIS buffer for 3×3 minutes. Sections were then blocked with MeOH and H_2_O_2_, washed with TRIS buffer, stained with a rabbit antibody against phospho-Akt (Ser473; R&D Systems) and subsequently counterstained with hematoxylin.

### Statistical Analysis

All data are expressed as mean values ±SEM. Statistical analyses were performed using the Student's *t* test or one-way ANOVA followed by a post hoc multiple comparison test where appropriate (SPSS software). Probability values >0.05 were considered significant.
